# Supplementary material for: USP8 inhibition reshapes an inflamed tumor microenvironment that potentiates the immunotherapy
Source: Nat Commun. 2022 Mar 31;13:1700. doi: 10.1038/s41467-022-29401-6 (PMC8971425; doi:10.1038/s41467-022-29401-6)
Supplement: Supplementary file 1 — Supplementary Information [file 41467_2022_29401_MOESM1_ESM.pdf]

Supplementary Information

USP8 inhibition reshapes an inflamed tumor microenvironment that potentiates the immunotherapy

Wenjun Xiong[1,2,11], Xueliang Gao[3,11], Tiantian Zhang[2], Baishan Jiang[2,4], Ming-Ming Hu[2,5], Xia Bu[6], Yang Gao[7,8], Lin-Zhou Zhang[2,9], Bo-Lin Xiao[2,9], Chuan He[1,2], Yishuang Sun[1,2], Haiou Li[2,10], Jie Shi[1,2], Xiangling Xiao[1,2], Bolin Xiang[1,2], Conghua Xie[1], Gang Chen[2,9], Haojian Zhang[2], Wenyi Wei[8], Gordon J. Freeman[6], Hong-Bing Shu[2,5], Haizhen Wang[3,*], Jinfang Zhang[1,2,*]

[1]Department of Radiation and Medical Oncology, Hubei Key Laboratory of Tumor Biological Behaviors, Hubei Cancer Clinical Study Center, Zhongnan Hospital of Wuhan University, Wuhan 430071, China
[2]Frontier Science Center for Immunology and Metabolism, Medical Research Institute, School of Medicine, Wuhan University, Wuhan 430071, China
[3]Department of Cell and Molecular Pharmacology & Experimental Therapeutics, Hollings Cancer Center, Medical University of South Carolina, Charleston, SC 29425, USA
[4]Center for Protein Degradation, Dana-Farber Cancer Institute, Harvard Medical School, Boston, MA 02115, USA
[5]Department of Infectious Diseases, Zhongnan Hospital of Wuhan University, Wuhan 430071, China
[6]Department of Medical Oncology, Dana-Farber Cancer Institute, Harvard Medical School, Boston, MA 02115, USA
[7]Department of Urology, The First Affiliated Hospital of Xi'an Jiaotong University, Xi'an, 710061 China
[8]Department of Pathology, Beth Israel Deaconess Medical Center, Harvard Medical School, Boston, MA 02115, USA
[9]The State Key Laboratory Breeding Base of Basic Science of Stomatology (Hubei-MOST) & Key Laboratory of Oral Biomedicine Ministry of Education and Department of Oral and Maxillofacial Surgery, School and Hospital of Stomatology, Wuhan University, Wuhan 430071, China
[10]Department of Dermatology, Zhongnan Hospital of Wuhan University, Wuhan 430071, China
[11]These authors contributed equally

[*]Corresponding authors: wangha@musc.edu (H.W.); jinfang_zhang@whu.edu.cn (J.Z.)

Supplementary Fig. 1

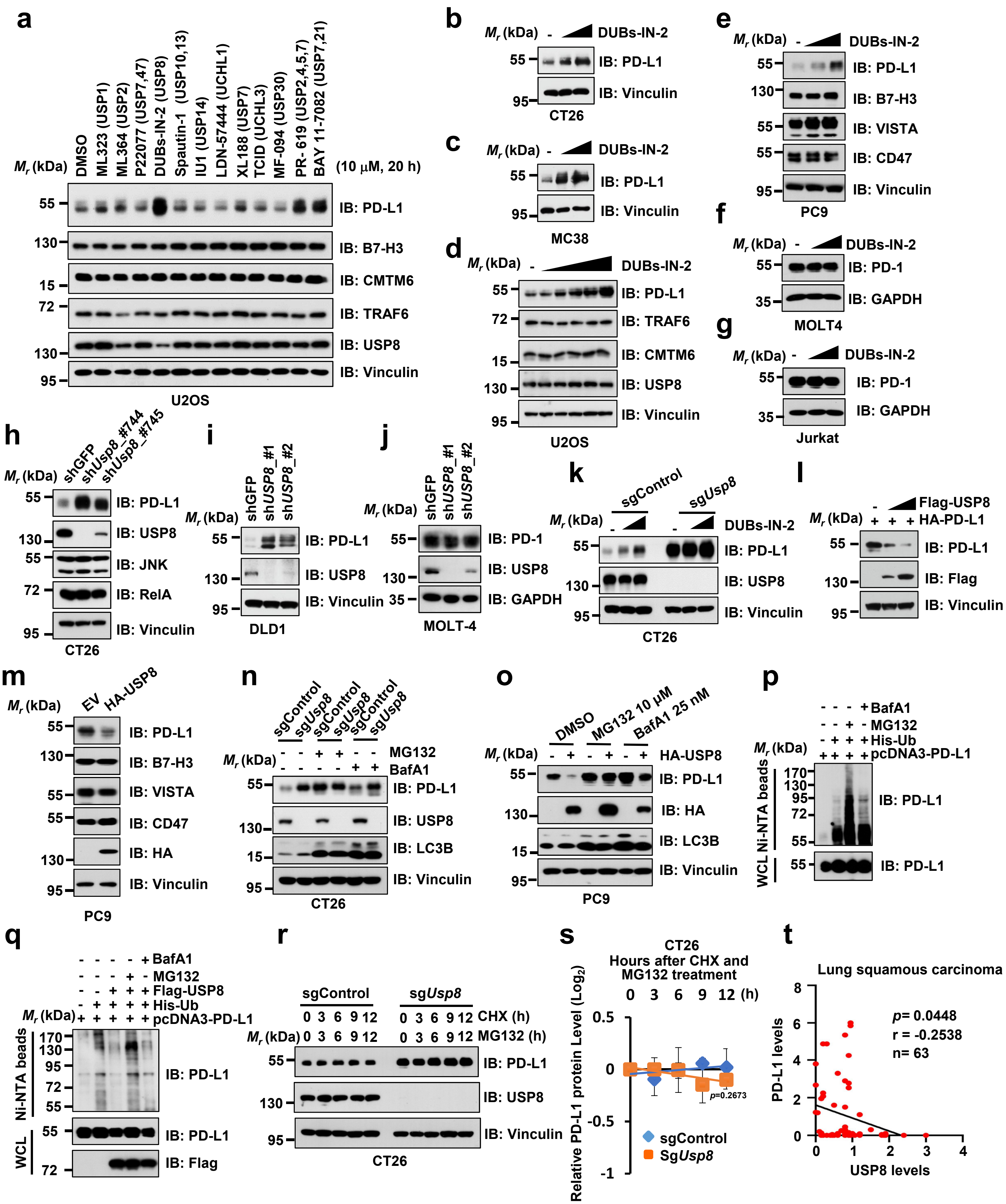

Supplementary Fig. 1. USP8 inhibition elevates PD-L1 protein abundance in cancer cells. **a** Immunoblot (IB) analysis of whole cell lysates (WCL) derived from U2OS cells treated with indicated inhibitors. **b-g** IB analysis of WCL derived from CT26 **(b)**, MC38 **(c)**, and U2OS **(d)**, PC9 **(e)**, MOLT-4 **(f)**, and Jurkat **(g)** cells treated with DUBs-IN-2 for 24 h. **h** IB analysis of WCL derived from sh*Usp8*- or shGFP-treated CT26 cells. **i**, **j** IB analysis of WCL derived from sh*Usp8*- or shGFP-treated DLD1 **(i)** or MOLT-4 **(j)** cells. **k** IB analysis of WCL derived from sgControl or sg*Usp8* CT26 cells treated with DUBs-IN-2 (4 μM and 6 μM) for 24 h. **l** IB analysis of WCL derived from 293T cells co-transfected with indicated constructs. **m** IB analysis of WCL derived from PC9 cells stably expressing empty vector (EV) or HA-USP8. **n** IB analysis of WCL derived from sgControl or sg*Usp8* CT26 cells treated with MG132 (10 μM) or BafA1 (100 nM) for 12 h. **o** IB analysis of WCL derived from EV or HA-USP8 PC9 cells treated with MG132 (10 μM) or BafA1 (25 nM) for 12 h. **p**, **q** IB analysis of WCL and Ni-NTA pull-down products derived from lysates of 293T cells transfected with indicated constructs. Cells were treated with 10 μM MG132 or 100 nM BafA1 for 12 h. **r**, **s** IB analysis of WCL derived from CT26 cells stably infected with indicated lentiviral sgRNAs. Cells were treated with 400 μg/ml cycloheximide (CHX) and MG132 (10 μM) at indicated time points **(r)**. PD-L1 band intensity was quantified by ImageJ, which was normalized to vinculin and then to the t = 0 time point **(s)**. Data were presented as mean ± S.D.; n = 3 biologically independent samples; Two-sided t-test. **t** Quantification of PD-L1 and USP8 staining intensities were performed by semi-quantitative scoring. n = 63, r = -0.2538, Correlation coefficients were calculated using the Pearson test. Two-sided *p*-value was given. For **a**-**q**, three independent biological repeats were conducted. Source data are provided as a Source Data file.

Supplementary Fig. 2

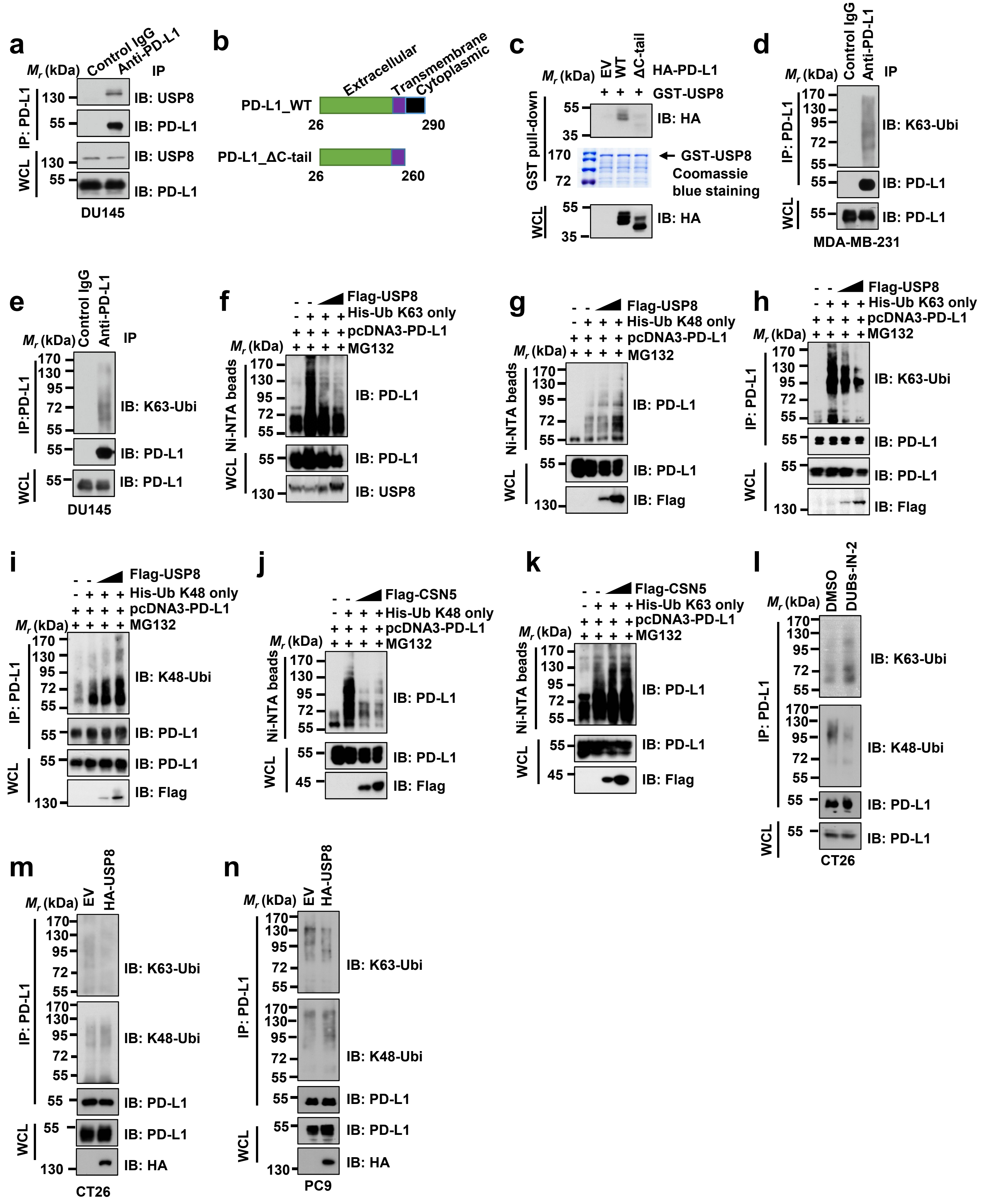

Supplementary Fig. 2. USP8 specifically removes the K63-linked poly-ubiquitination on PD-L1. a Immunoblot (IB) analysis of WCL and anti-PD-L1 IPs derived from DU145 cells. **b** A schematic illustration showing the different regions of PD-L1 protein. **c** IB analysis of glutathione S-transferase (GST) pull-down precipitates from 293T cell lysates with ectopic expression of HA-PD-L1 WT or HA-PD-L1 deleting C-tail (ΔC-tail) incubated with bacterially purified recombinant GST-USP8 protein. **d**, **e** IB analysis of WCL and anti-PD-L1 immunoprecipitations (IPs) derived from MDA-MB-231 (**d**) and DU145 (**e**). Cells were treated with 20 μM MG132 for 6 h. **f**, **g** IB analysis of WCL and Ni-NTA pull-down products derived from lysates of 293T cells transfected with Flag-USP8 and the indicated constructs. Cells were treated with 10 μM MG132 for 12 h. **h**, **i** IB analysis of WCL and anti-PD-L1 IPs derived from 293T transfected with USP8 and the indicated constructs. Cells were treated with 20 μM MG132 for 6 h. **j**, **k** IB analysis of WCL and Ni-NTA pull-down products derived from lysates of 293T cells transfected with Flag-CSN5 and the indicated constructs. Cells were treated with 10 μM MG132 for 12 h. **l** IB analysis of WCL and anti-PD-L1 IPs derived from CT26 treated with DUBs-IN-2 (10 μM) for 24 h. Cells were treated with 20 μM MG132 for 6 h. **m** IB analysis of WCL and anti-PD-L1 IPs derived from EV or HA-USP8 CT26 cells. Cells were treated with 20 μM MG132 for 6 h. **n** IB analysis of WCL and anti-PD-L1 IPs derived from EV or HA-USP8 PC9 cells. Cells were treated with 20 μM MG132 for 6 h. For **a**, **c**-**n**, three independent biological repeats were conducted. Source data are provided as a Source Data file.

Supplementary Fig. 3

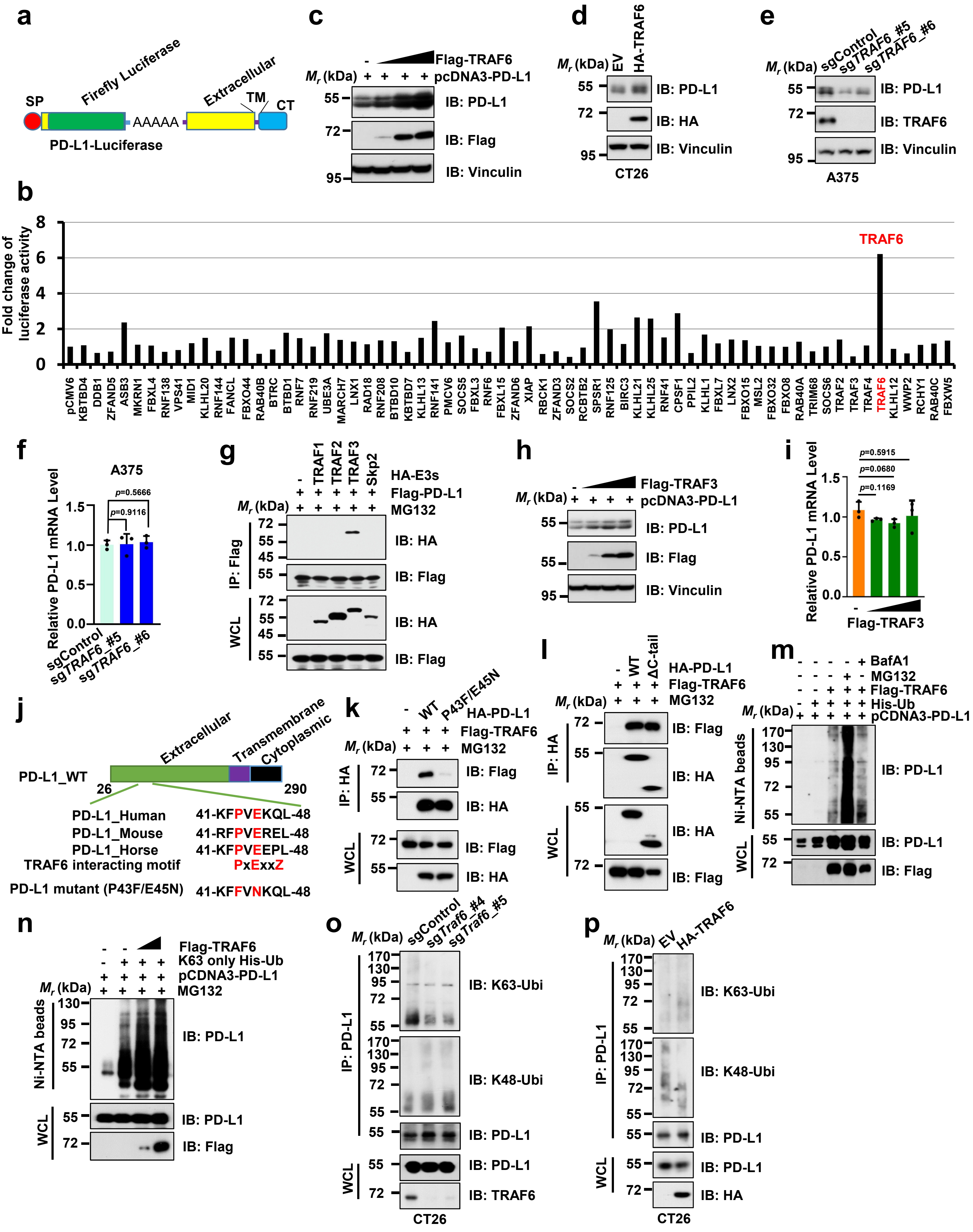

Supplementary Fig. 3. The E3 ligase TRAF6 positively regulates the PD-L1 protein abundance. a A schematic illustration of PD-L1-Luciferase fusion protein. PD-L1 contains signal peptide (SP), extracellular, transmembrane (TM), and cytoplasmic tail (CT). **b** Screening E3 ligase(s) for regulating PD-L1 stability through dual-luciferase reporter assay. The relative firefly luciferase activity was normalized to renilla luciferase activity and fold change was normalized to the control value of pCMV6. For E3 sub-library screening, n = one biologically independent sample. **c** Immunoblot (IB) analysis of whole cell lysates (WCL) derived from 293T cells transfected with indicated constructs. **d** IB analysis of WCL derived from CT26 cells stably expressing EV or HA-TRAF6. **e**, **f** IB analysis of WCL derived from sgGFP- or sg*TRAF6*-treated A375 cells (**e**). PD-L1 mRNAs were analyzed using RT-qPCR (**f**). **g** IB of WCL and anti-Flag immunoprecipitations (IPs) derived from 293T cells transfected with indicated constructs. **h**, **i** IB analysis of WCL derived from 293T cells co-transfected with indicated constructs **(h)**. PD-L1 mRNAs were analyzed using RT-qPCR **(i)**. **j** A schematic illustration showing potential TRAF6 interacting motif (PxExxZ, x: any amino acid residue, Z: aromatic or acidic residue) in PD-L1. **k** IB analysis of WCL and anti-HA IPs from 293T cells transfected with indicated constructs. **l** IB analysis of WCL and anti-HA IPs derived from 293T cells transfected with indicated constructs. **m**, **n** IB analysis of WCL and Ni-NTA pull-down products derived from lysates of 293T cells transfected with indicated constructs. **o** IB analysis of WCL and anti-PD-L1 IPs derived from WT or sg*Traf6* CT26 cells. **p** IB analysis of WCL and anti-PD-L1 IPs derived from EV or HA-TRAF6 CT26 cells. For **f** and **I**, Data were presented as mean ± S.D.; n = 3 biologically independent samples; Two-sided t-test. For **g**, **k**, **l**, **m**, and **n**, cells were treated with 10 μM MG132 or 100 nM BafA1 for 12 h. For **o** and **p**, Cells were treated with 20 μM MG132 for 6 h. For **c**, **d**, **g**, **k**-**p**, three independent biological repeats were conducted. Source data are provided as a Source Data file.

Supplementary Fig. 4

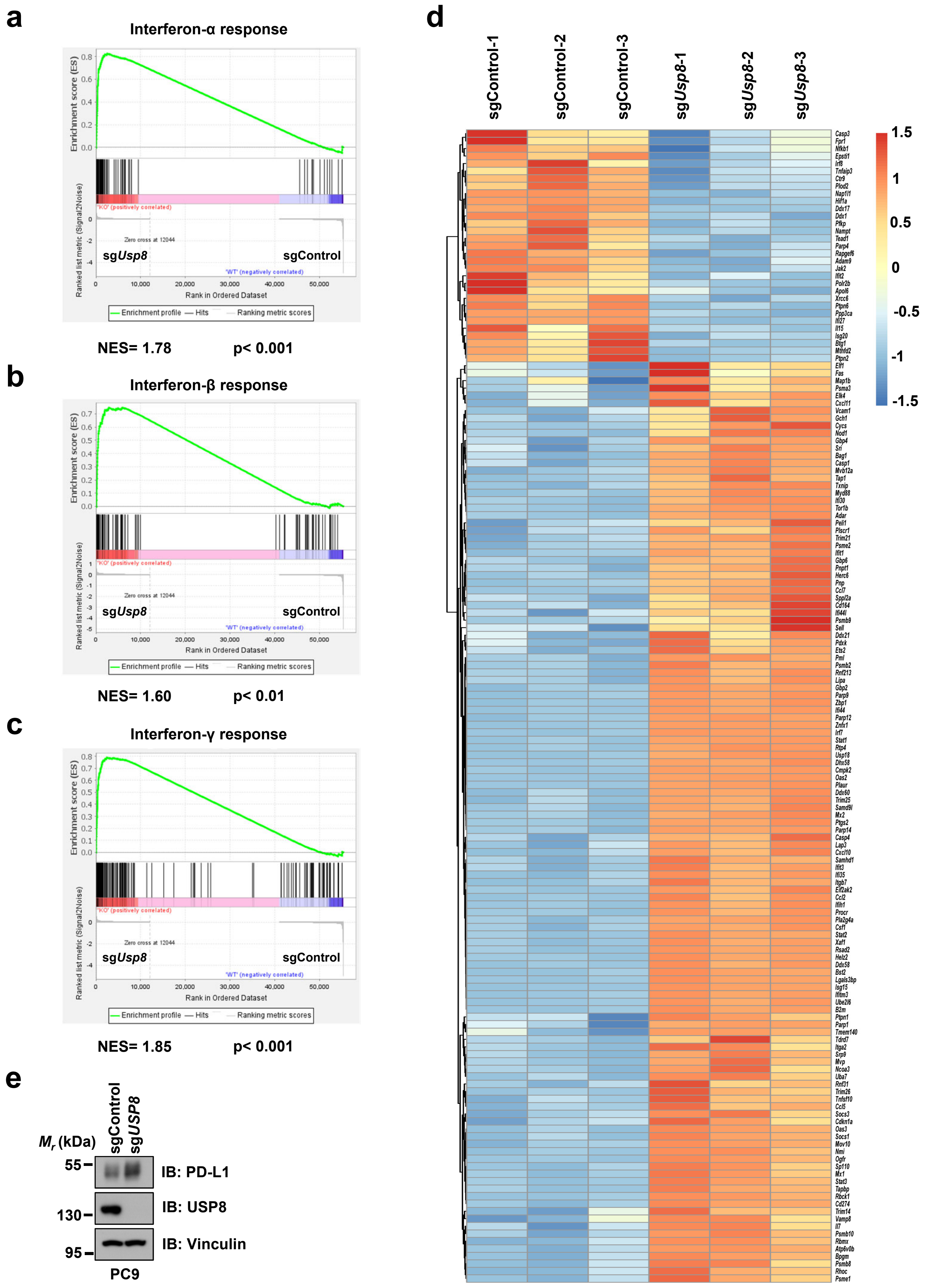

Supplementary Fig. 4. USP8 deficiency enriches interferon response genes. a Gene-set enrichment analysis (GSEA) for gene sets associated with interferon-α signaling pathway in sg*Usp8* versus sgControl cells. n = 3 biologically independent samples per group. *p* values are calculated using Kolmogorov-Smirnov tests. NES: normalized enrichment score. **b** GSEA for gene sets associated with interferon-β signaling pathway in sg*Usp8* versus sgControl cells. n = 3 biologically independent samples per group. *p* values are calculated using Kolmogorov-Smirnov tests. NES: normalized enrichment score. **c** GSEA for gene sets associated with interferon-γ signaling pathway in sg*Usp8* versus sgControl cells. n = 3 biologically independent samples per group. *p* values are calculated using Kolmogorov-Smirnov tests. NES: normalized enrichment score. **d** Heatmap showing differential expression of genes in the Supplementary Fig. 4a-c of interferon response signaling in GSEA analysis. **e** Immunoblot (IB) analysis of whole cell lysates (WCL) derived from PC9 cells infected with indicated lentiviral sgRNAs against *USP8* or control, which were selected with puromycin (1 μg/ml) and blasticidin (10 ug/ml) for generating stable cell lines before harvesting. Three independent biological repeats were conducted. Source data are provided as a Source Data file.

Supplementary Fig. 5

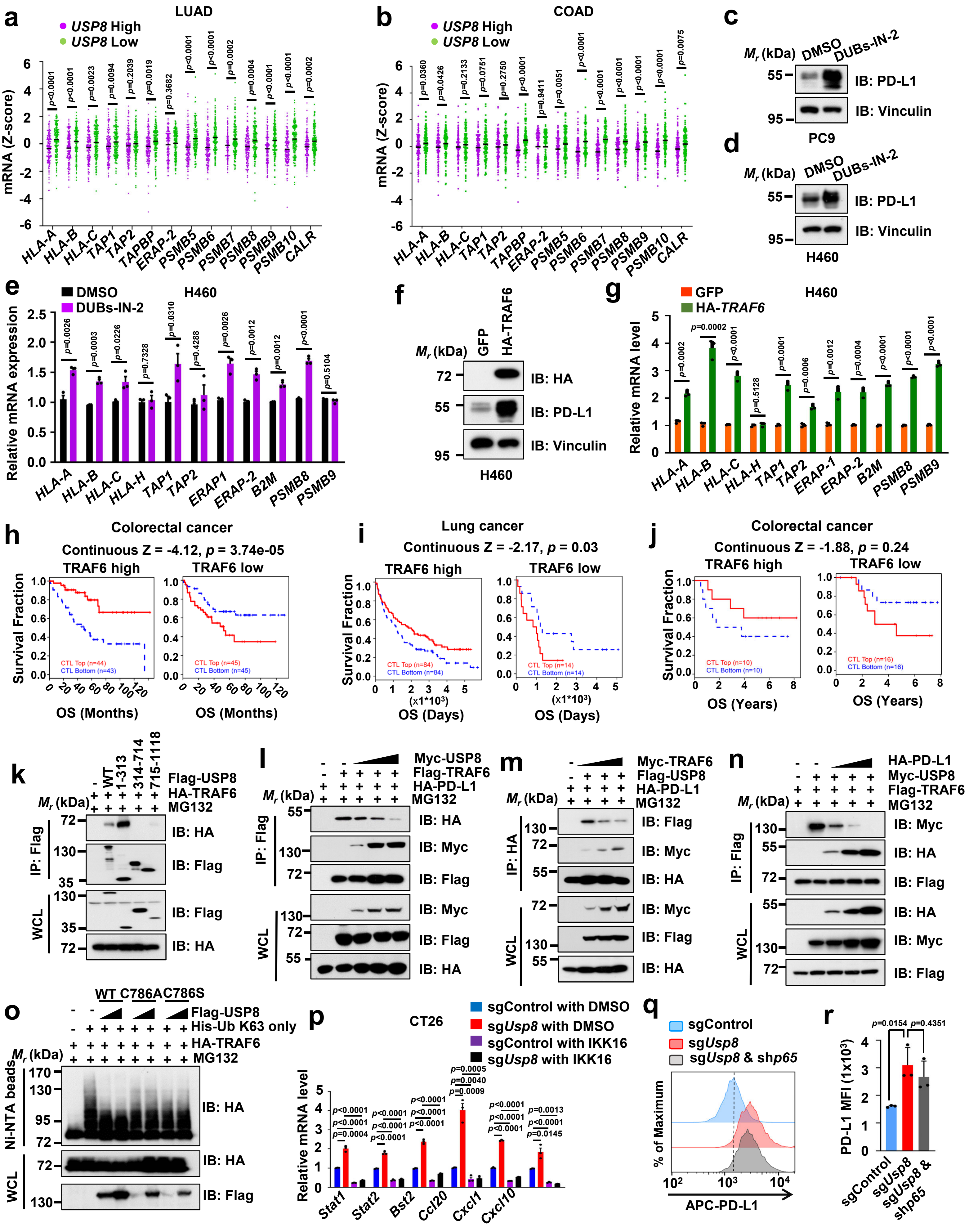

Supplementary Fig. 5. Inhibition of USP8 elevates the MHC-1 antigen presentation pathway largely through activating the TRAF6-NF-κB signaling. **a**, **b** mRNA levels of indicated genes in lung adenocarcinoma (LUAD) or colon adenocarcinoma (COAD) patients in TCGA cohort were analyzed under condition of *USP8* high (top 30%, n = 155 for LUAD or 85 for COAD) or low (bottom 30%, n = 155 for LUAD or 85 for COAD). Each circle represents a single sample, shown with the mean value of each group. Two-sided t-test. **c-e** IB analysis of WCL derived from PC9 treated with 2 μM DUBs-IN-2 (**c**) or H460 cells treated with 1 μM DUBs-IN-2 **(d)** for 24 h. mRNA levels of indicated genes from H460 cells were analyzed using RT-qPCR **(e)**. **f**, **g** IB analysis of WCL derived from H460 cells stably expressing GFP or TRAF6 **(f)**. mRNA levels of indicated genes were analyzed using RT-qPCR **(g)**. **h-j** The association between cytotoxic T lymphocyte level (CTL) and overall survival (OS) for colorectal cancer patients (GSE17536) **(h)**, lung cancer patients (GSE37745) **(i)** or colorectal cancer patients (GSE71187) **(j)** under condition of *TRAF6* high or low expression was analyzed using Kaplan-Meier curves by Tumor Immune Dysfunction and Exclusion (TIDE) algorithm. Two-sided Wald test. **k-n** IB analysis of WCL and anti-Flag or anti-HA IPs derived from 293T cells transfected with indicated constructs. Cells were treated with 10 μM MG132 for 12 h. **o** IB analysis of WCL and Ni-NTA pull-down products derived from lysates of 293T cells transfected with indicated constructs. Cells were treated with 10 μM MG132 for 12 h. **p** mRNA levels of indicated genes from sgControl or sg*Usp8* CT26 treated with 10 μM IKK16 for 15 h. **q**, **r** Cell surface $H2K^d/H2D^d$ was analyzed by flow cytometry of indicated CT26 cells. For **e**, **g**, **p**, and **r**, data were presented as mean ± S.D.; n = 3 biologically independent samples; Two-sided t-test. For **c**, **d**, **f**, **k-o**, three independent biological repeats were conducted. Source data are provided as a Source Data file.

Supplementary Fig. 6

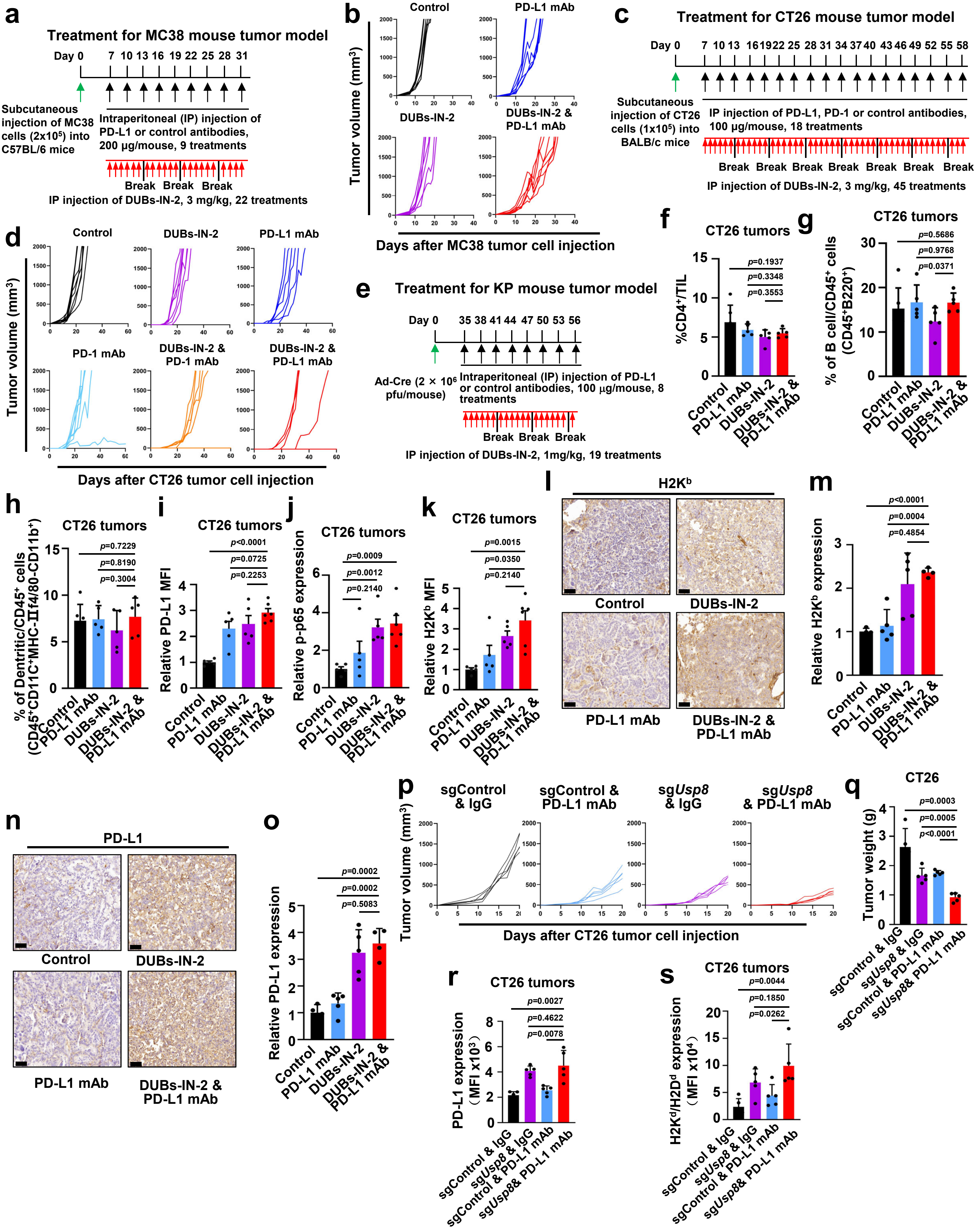

Supplementary Fig. 6. USP8 inhibitor enhances the therapeutic effect of anti-PD-1/PD-L1 treatment. a A schematic treatment plan for C57BL/6 mice bearing subcutaneous MC38 tumors. **b** Tumor volumes of C57BL/6 mice bearing MC38 cells with indicated treatment were measured every three days and plotted individually. n = 7 mice/group. **c** A schematic treatment plan for BALB/c mice bearing subcutaneous CT26 tumors. **d** Tumor volumes of BALB/c mice bearing CT26 cells with indicated treatments measured every three days and plotted individually. n = 9 (control), 7 (DUBs-IN-2), 6 (PD-L1 mAb), 7 (PD-1 mAb), 8 (PD-1 mAb plus DUBs-IN-2) or 7 (PD-L1 mAb plus DUBs-IN-2) mice. **e** A scheme of combinational therapy for $Kras^{G12D/+}Tp53^{fl/fl}$ (KP) mice bearing autochthonous lung cancers. **f-h** Quantification of flow cytometry result of tumor infiltrating $CD4^{+}$ T cells **(f)**, B cells **(g)**, or Dentritic cells **(h)** in CT26 tumors after indicated treatments. n = 6, 5, 5, or 5 mice/group. Data were presented as mean ± S.D.; Two-sided t-test. **i-k** Relative mean fluorescence intensity (MFI) of PD-L1 **(i)**, p-p65 **(j)**, or $H2K^{b}$ **(k)** on CT26 tumor cells after indicated treatments. n = 5, 5, 5, or 6 mice/group. Data were presented as mean ± S.D.; Two-sided t-test. **l-o** Representative images from IHC staining of $H2K^{b}$ (**l**) or PD-L1 (**n**) in tissues of tumor-burdened lungs of KP mice. Scale bar: 50 µm. Relative of $H2K^{b}$ (**m**) or PD-L1 (**o**) staining intensities were performed by semi-quantitative scoring. n = 4, 5, 5, or 4 mice/group. Data were presented as mean ± S.D.; Two-sided t-test. **p**, **q** Tumor volumes of C57BL/6 mice bearing sgControl or sg*Usp8* CT26 cells treated with indicated treatment were measured every two days and plotted individually (**p**). Quantification of tumor weight of sgControl or sg*USP8* CT26 tumors after indicated treatments. n = 5 mice/group (**q**). Data were presented as mean ± S.D.; Two-sided t-test. **r**, **s** Quantification for MFI of PD-L1 **(r)** or $H2K^{d}/H2D^{d}$ **(s)** on sgControl or sg*USP8* CT26 tumor cells after indicated treatments. n = 5 mice/group. Data were presented as mean ± S.D.; Two-sided t-test. Source data are provided as a Source Data file.

Supplementary Fig. 7

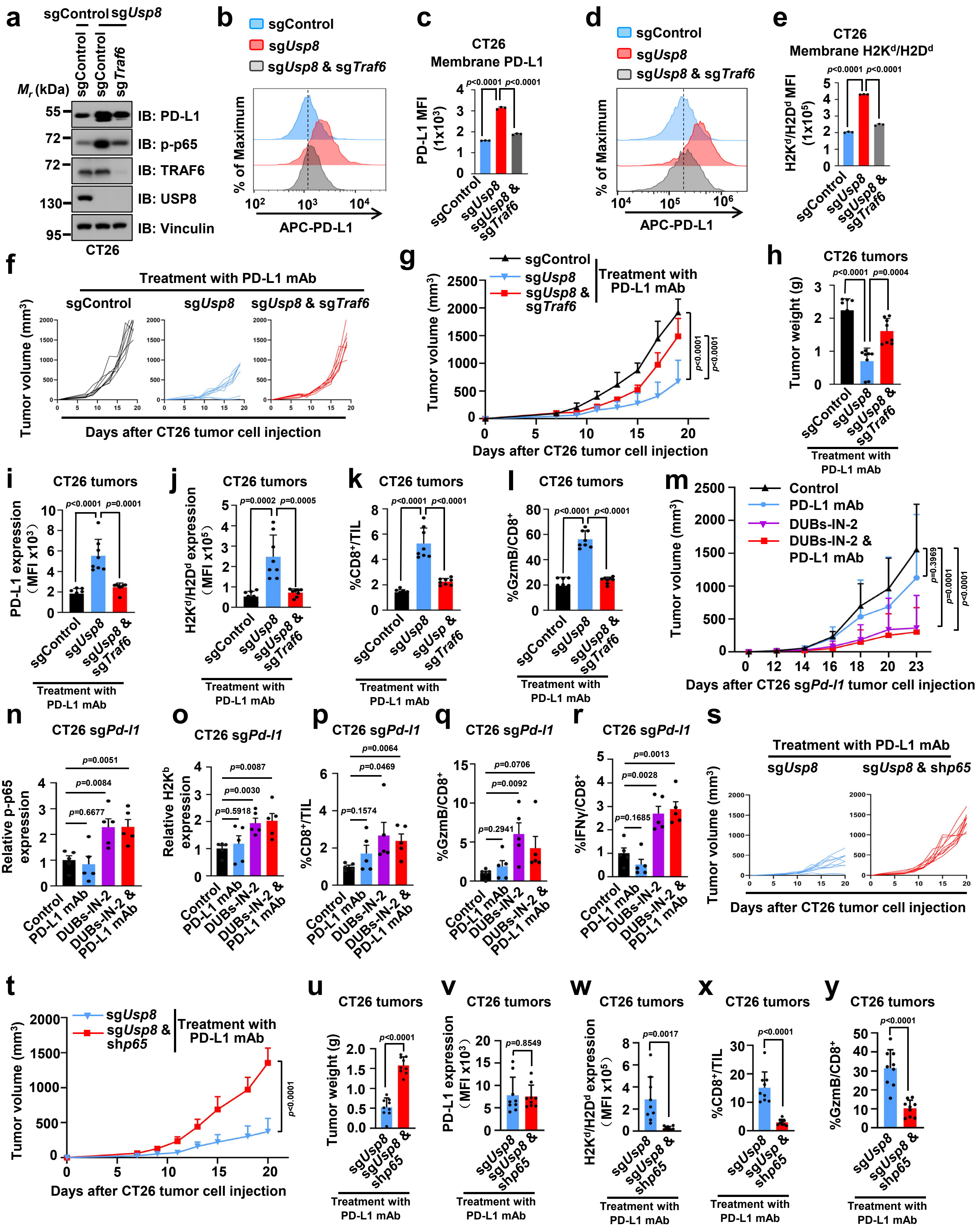

Supplementary Fig. 7. Deficiency of *Pd-l1* or *p65* limited tumors sensitive to anti-PD-L1 immunotherapy. a-e Immunoblot (IB) analysis of whole cell lysates (WCL) derived from sgControl or sg*Usp8* CT26 cells infected with indicated lentiviral sgControl or sg*TRAF6* (**a**). Cell surface PD-L1 (**b**) or H2K^d/H2D^d (**d**) on indicated CT26 cells was analyzed. Quantification for mean fluorescence intensity (MFI) of surface PD-L1 (**c**) or H2K^d/H2D^d (**e**) on indicated CT26 tumor cells. Data were presented as mean ± S.D.; n = 3 biologically independent samples; Two-sided t-test. **f**, **g** Tumor growth curve of C57BL/6 mice bearing sgControl, sg*Usp8* or sg*Usp8* & sg*Traf6* CT26 cells treated with PD-L1 mAb. n = 8 mice/group. **h** Tumor weight of indicated CT26 tumors with different treatments. **i**, **j** Quantification of surface PD-L1 **(i)** or H2K^d/H2D^d **(j)** on indicated CT26 tumor cells with different treatments. **k, l** Quantification of tumor-infiltrating CD8$^+$ T **(k)** cells or GzmB **(l)** in indicated CT26 tumors after different treatments. **m** Tumor growth curve of BALB/c mice bearing sg*Pd-l1* CT26 cells with indicated treatment. n = 5, 6, 6 or 6 mice/group. **n**, **o** Relative MFI of p-p65 **(n)** or H2K^b **(o)** in sg*Pd-l1* CT26 tumor cells after indicated treatments. **p-r** Quantification of tumor-infiltrating CD8$^+$ T cells **(p)**, GzmB **(q)** and IFNγ **(r)** in sg*Pd-l1* CT26 tumors after indicated treatments. **s**, **t** Tumor volumes or growth curve of C57BL/6 mice bearing sg*Usp8* or sg*Usp8* & sh*p65* CT26 cells were treated with PD-L1 mAb. n = 9 mice/group. **u** Quantification of tumor weight of sg*Usp8* or sg*Usp8* & sh*p65* CT26 tumors after indicated treatments. **v**, **w** Quantification for MFI of surface PD-L1 **(v)** or H2K^d/H2D^d **(w)** on sg*Usp8* or sg*Usp8* & sh*p65* CT26 tumor cells after indicated treatment. **x**, **y** Quantification of tumor-infiltrating CD8$^+$ T cells **(x)** or GzmB **(y)** in sg*Usp8* or sg*Usp8* & sh*p65* CT26 tumors after indicated treatments. For **g**, **m**, **t**, data were presented as mean ± S.D.; two-way ANOVA test. For **h**-**l**, n = 8 mice/group; for **n-r**, n = 5 mice/group; for **u-y**, n = 9 mice/group; data were presented as mean ± S.D.; two-sided *t*-test. Source data are provided as a Source Data file.

Supplementary Fig. 8

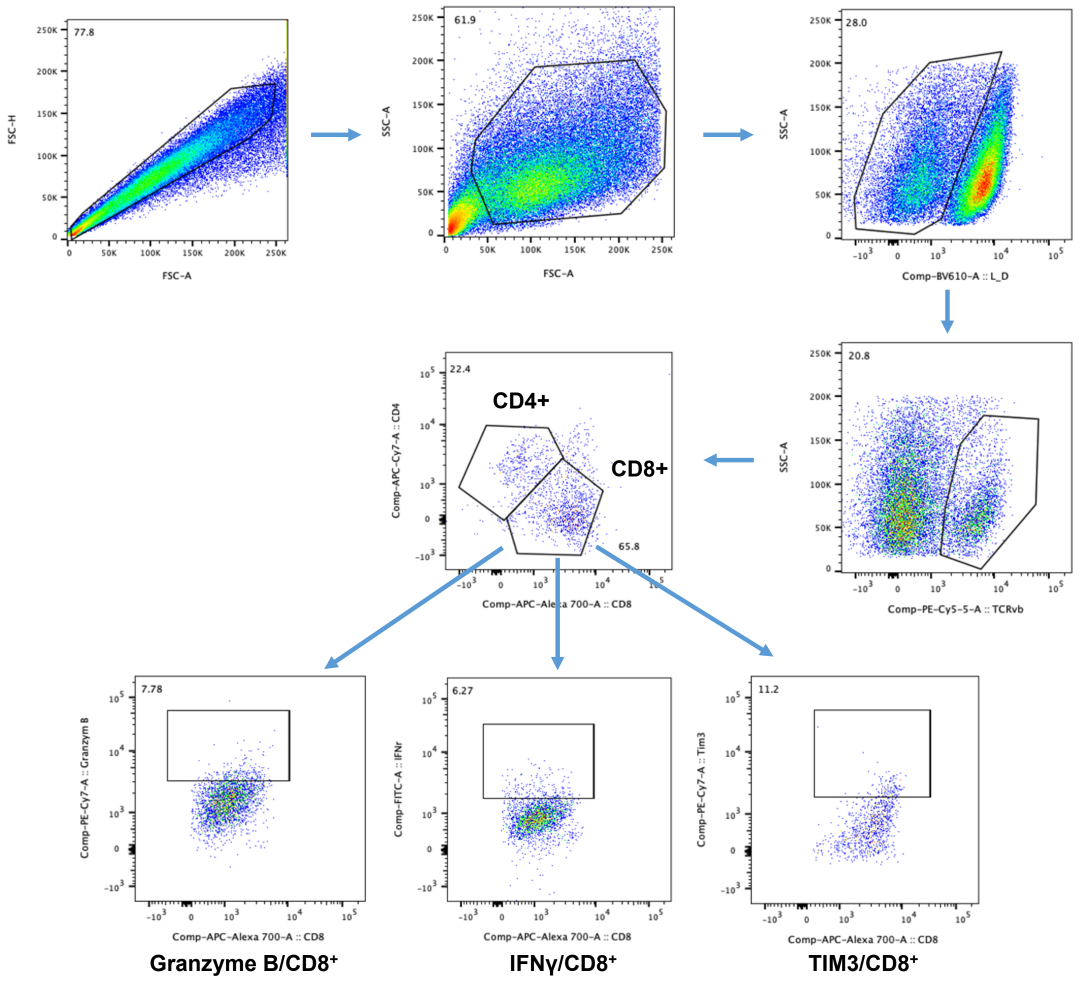

Supplementary Fig. 8. Exemplifying the gating strategy of flow cytometry analysis. Forward versus side scatter (FSC vs SSC) gating was used to identify cells and exclude cell debris and dead cells. A forward scatter (FCS-H) vs. forward scatter area (FCS-A) density plot was used to exclude doublets. In some experiments, dead cells were further gated out on SSC-A and live/dead staining. Cytokine expression including Granzyme B and IFNγ were detected in the populations of $CD8^{+}$cells.

Supplementary Table 1

qPCR primers of human genes		
Name	***Primer-F***	***Primer-R***
hPD-L1	5-TGGCATTTGCTGAACGCATTT-3	5-TGCAGCCAGGTCTAATTGTTTT-3
hBst2	5-CACACTGTGATGGCCCTAATG-3	5-GTCCGCGATTCTCACGCTT-3
hIrgm	5-CCTCACCTCCTACTGAGCTG-3	5-GTTTTGGCAAGCATCACATGATT-3
hStat1	5-CAGCTTGACTCAAAATTCCTGGA-3	5-TGAAGATTACGCTTGCTTTTCCT-3
hStat2	5-CCAGCTTTACTCGCACAGC-3	5-AGCCTTGGAATCATCACTCCC-3
hIrf7	5-GTGGACTGAGGGCTTGTAG-3	5-TCAACACCTGTGACTTCATGT-3
hIfit1	5-AGAAGCAGGCAATCACAGAAAA-3	5-CTGAAACCGACCATAGTGGAAAT-3
hCasp1	5-TTTCCGCAAGGTTCGATTTTCA-3	5-GGCATCTGCGCTCTACCATC-3
hCcl20	5-TGCTGTACCAAGAGTTTGCTC-3	5-CGCACACAGACAACTTTTTCTTT-3
hMyd88	5-GGCTGCTCTCAACATGCGA-3	5-CTGTGTCCGCACGTTCAAGA-3
hOas1	5-TGTCCAAGGTGGTAAAGGGTG-3	5-CCGGCGATTTAACTGATCCTG-3
hOas2	5-AGGTGGCTCCTATGGACGG-3	5-TTTATCGAGGATGTCACGTTGG-3
hOas3	5-TCTGAGACTCACGTTTCCTGA-3	5-CACTGTTGAGGAGGGTAGAGTA-3
hIsg15	5-CGCAGATCACCCAGAAGATCG-3	5-TTCGTCGCATTTGTCCACCA-3
hIfit3	5-TCAGAAGTCTAGTCACTTGGGG-3	5-ACACCTTCGCCCTTTCATTTC-3
Hifitm1	5-CCAAGGTCCACCGTGATTAAC-3	5-ACCAGTTCAAGAAGAGGGTGTT-3
hIFIT2	5-AAGCACCTCAAAGGGCAAAAC-3	5-TCGGCCCATGTGATAGTAGAC-3
hIFIT5	5-TGGAGCCTGACAATCCAGAAT-3	5-TGGGATGATATTTGGTCCAGGA-3
hsp100	5-AAGGCTGAGCCAACAGAGTC-3	5-ATATCCACCAGTCGCACAGAA-3
hIL6	5-ACTCACCTCTTCAGAACGAATTG-3	5-CCATCTTTGGAAGGTTCAGGTTG-3
hHLA-A	5-ACCCTCGTCCTGCTACTCTC-3	5-CTGTCTCCTCGTCCCAATACT-3
hHLA-B	5-CAGTTCGTGAGGTTCGACAG-3	5-CAGCCGTACATGCTCTGGA-3
hHLA-C	5-GGACAAGAGCAGAGATACACG-3	5-CAAGGACAGCTAGGACAACC-3
hB2M	5-GAGGCTATCCAGCGTACTCCA-3	5-CGGCAGGCATACTCATCTTTT-3
hTAP1	5-CTGGGGAAGTCACCCTACC-3	5-CAGAGGCTCCCGAGTTTGTG-3
hTAP2	5-TGGACGCGGCTTTACTGTG-3	5-GCAGCCCTCTTAGCTTTAGCA-3
hERAP1	5-CCCCTCAAATGGTCCCTTGC-3	5-GAGATGCTTCAGTGCTCTGAC-3
hERAP2	5-CACTAATGGGGAACGATTTCCTT-3	5-CTGACCAAGACTTCGATCTTCTC-3
hPSMB8	5-TCTCCAGAGCTCGCTTTACC-3	5-CACTCCATGCTGGAACTTGA-3
hPSMB9	5-CGTTGTGATGGGTTCTGATTCC-3	5-GACAGCTTGTCAAACACTCGGTT-3
hHLA-H	5-GTCTGGCACCCTAGTCATTG-3	5-ACGTTCAGCTAAGACGTAGTGC-3
hGAPDH	5-GGAGCGAGATCCCTCCAAAAT-3	5-GGCTGTTGTCATACTTCTCATGG-3

Continued for Supplementary Table 1

qPCR primers of mouse genes		
mPd-l1	5-GCTCCAAAGGACTTGTACGTG-3	5-TGATCTGAAGGGCAGCATTTC-3
mIfitm3	5-CCCCCAAACTACGAAAGAATCA-3	5-ACCATCTTCCGATCCCTAGAC-3
mBst2	5-TGTAGAGACGGGTTGCGAG-3	5-CAGGGACTCCTGAAGGGTC-3
mIrgm-1	5-AGACCCATTATGCTCCCCTGA-3	5-CGGTGCTCCTACTGACCTCA-3
mIrgm2	5-TCTCCGACGCTGTATTCATTCC-3	5-CTTCTTTCACGGCAGTCTCAAT-3
mStat1	5-GCTGCCTATGATGTCTCGTTT-3	5-TGCTTTTCCGTATGTTGTGCT-3
mStat2	5-GTTACACCAGGTCTACTCACAGA-3	5-TGGTCTTCAATCCAGGTAGCC-3
mIrf7	5-TCCAGTTGATCCGCATAAGGT-3	5-CTTCCCTATTTTCCGTGGCTG-3
mIfit1	5-ATCGCGTAGACAAAGCTCTTC-3	5-GTTTCGGGATGTCCTCAGTTG-3
mIfi202b	5-TGGGTCTCTGGCAATACATGA-3	5-CTCCATCAACCAGCCTTTCTTT-3
mIfi203	5-AAAAGAGGAAGATTGCCTCCAG-3	5-CTTGGTGCCTTGTTTGAGGAA-3
mIfi205	5-AAGATCAAGGCATCTGGGAAAG-3	5-CCTCTGGGAATGTTCTGGTTC-3
mCasp1	5-AATACAACCACTCGTACACGTC-3	5-AGCTCCAACCCTCGGAGAAA -3
mCcl20	5-ACTGTTGCCTCTCGTACATACA-3	5-GAGGAGGTTCACAGCCCTTTT-3
mMyd88	5-ATCGCTGTTCTTGAACCCTCG-3	5-CTCACGGTCTAACAAGGCCAG-3
mOas1a	5-GCCTGATCCCAGAATCTATGC-3	5-GAGCAACTCTAGGGCGTACTG-3
mOas1b	5-GGGCCTCTAAAGGGGTCAAG-3	5-TCAAACTTCACTCCACAACGTC-3
mOas1c	5-GTATGCTGAACCCCAATTCTACA-3	5-TGGCTGTGGTTACTTTTTCTTGA-3
mOas3	5-TCTGGGGTCGCTAAACATCAC-3	5-GGCAATCCTTATCACTCTTGGTC-3
mIsg15	5-GGTGTCCGTGACTAACTCCAT-3	5-CTGTACCACTAGCATCACTGTG-3
mCxcl10	5-CCAAGTGCTGCCGTCATTTTC-3	5-TCCCTATGGCCCTCATTCTCA-3
mCxcl1	5-ACTGCACCCAAACCGAAGTC-3	5-TGGGGACACCTTTTAGCATCTT-3
mIfit3	5-CCTACATAAAGCACCTAGATGGC-3	5-ATGTGATAGTAGATCCAGGCGT-3
mIl6	5-CTGCAAGAGACTTCCATCCAG-3	5-AGTGGTATAGACAGGTCTGTTGG-3
mTlr3	5-GTGAGATACAACGTAGCTGACTG-3	5-TCCTGCATCCAAGATAGCAAGT-3
mPsmb6	5-GGACAACCACTGGGTCCTAC-3	5-CAAGCTGGTAAGTGACAGCGT-3
mPsmb7	5-GTGTCGGTGTTTCAGCCAC-3	5-GTGCCAGTTTTCCGAGCTTTC-3
mPsmb8	5-GTGCAGGTTGTATTATCTTCGGA-3	5-CGAGTCCCATTGTCATCTACG-3
mPsmb10	5-GAGGAATGCGTCCTTGGAACA-3	5-CACAACCGAATCGTTAGTGGC-3
H2-K1	5-GCTGGTGAAGCAGAGAGACTCAG-3	5-GGTGACTTTATCTTCAGGTCTGCT-3
H2-D1	5-AGTGGTGCTGCAGAGCATTACAA-3	5-GGTGACTTCACCTTTAGATCTGGG-3
mB2m	5-TGGTGCTTGTCTCACTGACC-3	5-TTCAGTATGTTCGGCTTCCC-3
mTap1	5-AGTCTGGAGCCCACGATTTCATC-3	5-GGGTGATAAGAAGAACCGTCCG-3
mTapbp	5-GGCCTGTCTAAGAAACCTGCC-3	5-CCACCTTGAAGTATAGCTTTGGG-3
mErap1	5-TAATGGAGACTCATTCCCTTGGA-3	5-AAAGTCAGAGTGCTGAGGTTTG-3
mGapdh	5-AGGTCGGTGTGAACGGATTTG-3	5-GGGGTCGTTGATGGCAACA-3
